# A short CD3/CD28 costimulation combined with IL-21 enhance the generation of human memory stem T cells for adoptive immunotherapy

**DOI:** 10.1186/s12967-016-0973-y

**Published:** 2016-07-19

**Authors:** C. Alvarez-Fernández, L. Escribà-Garcia, S. Vidal, J. Sierra, J. Briones

**Affiliations:** Hematology Service, Hospital de la Santa Creu i Sant Pau, Mas Casanovas 90, 08041 Barcelona, Spain; Autonomous University, Barcelona, Spain; IIB-Institut Recerca Hospital de la Santa Creu I Sant Pau, Barcelona, Spain; Laboratory of Experimental Hematology–IIB, Institut Recerca Hospital de la Santa Creu I Sant Pau, Barcelona, Spain

**Keywords:** Immunotherapy, IL-21, Memory stem T cell

## Abstract

**Background:**

Immunotherapy based on the adoptive transfer of gene modified T cells is an emerging approach for the induction of tumor-specific immune responses. Memory stem T cells, due to their enhanced antitumor and self-renewal capacity, have become potential candidate for adoptive T cell therapy of cancer. Methods to generate memory stem T cells ex vivo rely on CD3/CD28 costimulation and the use of cytokines such as IL-7 and IL-15 during the entire culture period. However, a strong costimulation may induce differentiation of memory stem T cells to effector memory T cells. Here we show that manipulation of the length of the costimulation and addition of IL-21 enhance the ex vivo expansion of memory stem T cells.

**Methods:**

Purified naïve T cells from healthy donors were cultured in the presence of anti-CD3/CD28 coated beads, IL-7, IL-15 and/or IL-21 (25 ng/ml). T cells phenotype from the different memory and effector subpopulations were analyzed by multiparametric flow cytometry.

**Results:**

A short anti-CD3/CD28 costimulation of naïve T cells, combined with IL-7 and IL-15 significantly increased the frequencies of CD4^+^ and CD8^+^ memory stem T cells ex vivo, compared to a prolonged costimulation (34.6 ± 4.4 % vs 15.6 ± 4.24 % in CD4^+^; p = 0.008, and 20.5 ± 4.00 % vs 7.7 ± 2.53 % in CD8^+^; p = 0.02). Moreover, the addition of IL-21 to this condition further enhanced the enrichment and expansion of CD4^+^ and CD8^+^ memory stem T cells with an increase in the absolute numbers (0.7 × 10^6^ ± 0.1 vs 0.26 × 10^6^ ± 0.1 cells for CD4^+^; p = 0.002 and 1.1 × 10^6^ ± 0.1 vs 0.27 × 10^6^ ± 0.1 cells for CD8^+^; p = 0.0002; short + IL-21 vs long).

**Conclusions:**

These new in vitro conditions increase the frequencies and expansion of memory stem T cells and may have relevant clinical implications for the generation of this memory T cell subset for adoptive cell therapy of patients with cancer.

**Electronic supplementary material:**

The online version of this article (doi:10.1186/s12967-016-0973-y) contains supplementary material, which is available to authorized users.

## Background

Adoptive T cell therapy (ACT) represents a highly promising strategy to treat cancer [1, 2]. While this approach has been mostly based on the use of terminally differentiated effector T cells, recent studies indicate that the use of less differentiated T cells with extensive replicative capacity has greater engraftment and antitumor effect [3–6]. In particular, the recently described memory stem T cells (T_SCM_), defined as CD62L^+^CCR7^+^CD45RA^+^CD95^+^CD45RO^−^CD28^+^CD27^+^IL2Rb^+^IL7Rα^+^, have enhanced proliferation, self-renewal, persistence and are able to differentiate into central memory, effector memory and effector T cells [6, 7]. Remarkably, studies in murine models have shown that T_SCM_ have greater antitumor effect in vivo compared to other more differentiated T cell subsets [6, 8].

All these features make T_SCM_ a promising T cell subset candidate for ACT of cancer. Although T_SCM_ have been identified in the peripheral blood under basal conditions, the very low amount of these cells pose difficulty to their isolation, limiting their use for ACT of patients with cancer. Gattinoni et al. showed that CD8^+^ T_SCM_ could be generated through the induction of the Wnt-β-catenin signaling [6]. Further studies demonstrated the generation and expansion of CD8^+^ T_SCM_ from naïve T cells [9–11] through CD3/CD28 costimulation in combination with IL-7 and IL-15 cytokines. However, prolonged in vitro costimulation decrease the expression of memory markers substantially (e.g., CD62L, CCR7 or CD27) leading to a swift to more differentiated T cells [12, 13]. For these reasons, identification of reproducible methods to generate and expand large numbers of T_SCM_ for ACT of cancer remains a clinical priority.

IL-21, the most recent member of the common γ-chain (γc) receptor cytokine family (that includes IL-2, IL-7, and IL-15), has a role in innate and adaptive immunity [12], and contrary to IL-2, plays a key role in the development and maintenance of central memory T cells by the induction of an early differentiation phenotype [14]. Importantly, T cells generated under IL-21 showed a superior antitumor effect in vivo in experimental models [15–17]. IL-21 acts synergistically with IL-7 and IL-15 to promote proliferation and survival of both memory and naïve T cells [18, 19]. However, to our knowledge the effects of IL-21 on the T_SCM_ generation have not been studied so far.

We explored whether manipulating the length of CD3/CD28 costimulation and/or the addition of IL-21 could enrich for the T_SCM_ population within T cells cultured under IL-7 and IL-15. We found that both, a very short costimulation and the addition of IL-21 increase the frequencies of CD4^+^ and CD8^+^ T_SCM_ and enhance their proliferative capacity, resulting in a significant increase of T_SCM_ numbers at the end of the culture. Our work reveals a new method to ex vivo enrich and expand T_SCM_, which may have relevant clinical implications for ACT of patients with cancer.

## Methods

### T-cell isolation, sorting and culture

Peripheral blood mononuclear cells (PBMCs) were obtained from healthy donors (n = 6) after informed consent, isolated by centrifugation through a ficoll-hypaque gradient (lymphoprep, Axis-shield, UK), and frozen until use. Naïve T-cells were enriched with an on demand naïve T-cell enrichment kit (Stem cell technologies, Canada) including CD14, CD16, CD19, CD20, CD36, CD45RO, CD56, CD66b, CD57, CD94, CD123, CD244, TCRγ/δ, glycophorin A and dextran-coated magnetic particles and according to manufacturer’s instructions.

T_SCM_, T_CM_, and T_EM_ were FACS-purified on a FACS CANTO II cell sorter (BD Bio- sciences, USA) using the fluorescent antibodies anti-CD4, anti-CD8, anti-CD45RA, anti-CD45RO, anti-CD27, anti-CD95 and anti-CCR7.

Primary cells were cultured in RPMI-1640 medium supplemented with 10 % heat inactivated fetal bovine serum (FBS), penicillin (100 U/ml) and streptomycin (100 µg/ml) (ThermoFisher Scientific, USA). All cell cultures were performed at 37 °C in a fully humidified atmosphere with 5 % CO_2_ in air.

Purified naïve T cells (higher than 95 %) were activated with anti-CD3/CD28 magnetic beads (Life Technologies, USA) in 1:2 bead/T-cell ratio, and then cultured with: IL-7 and IL-15 at 25 ng/ml each [11] (Peprotech, USA) or IL-7, IL-15 and IL-21 at 25 ng/ml each (Peprotech, USA). 2,5 × 10^5^ cells/ml per well were plated in a 24-wells plate. After day 6, cells were cultured at a concentration of 1 × 10^6^ cells/ml and plated in 12 or 6-well plates (depending on cell number). Cytokines and medium were replaced every 3–4 days, and cells were counted every 3–4 days by Trypan blue dye exclusion.

All donor’s samples were obtained according to a protocol approved by the Ethics Comittee of Hospital Santa Creu I Sant Pau.

### Lentivirus production and transduction

A second generation lentival expression vector pLS-CG was used to generate GFP-encoding lentivirus. Lentivirus was produced in 293T cells cotransfected with the lentiviral vector and the packaging vectors pCMV-dR8.91 and pCMV-G using jet PRIME (Polyplus Transfection). Medium was changed 4 h after transfection, and lentivirus collected and concentrated after 48–72 h. Transduction was performed 48 h after initial stimulation by 1000 g spinoculation for 2 h at 32 °C in the presence of polybrene (5 μg/ml) (Sigma-Aldrich, USA). GFP expression was detected by flow cytometry in T_SCM_ at day 8 of culture.

### Flow cytometry, and antibodies and intracellular staining

Cells were thawed and labeled with fluorescent antibodies against human CD4-APC-Vio-770 (clone VIT4), CD8-Viogreen (clone BW135/80), CD45RA-PerCP-Vio700 (clone T6D11), CD45RO-PE (clone UCHL1), CD27-Vioblue (clone M-T271), CD95-FITC (clone DX2) and CCR7-PE-vio770 (clone REA108) (all from Miltenyi Biotech, Germany).

For intracellular staining, cells were stimulated with 50 ng/mL PMA and 1 μg/ml ionomycin (Sigma-Aldrich, USA) during 6 h. Brefeldin 1× (eBioscience, USA) was added for the last 4 h. Cells were stained with surface antibodies, washed and fixed with BD Cytofix/Cytoperm (BD Biosciences, USA), and intracellular staining was performed with anti–IL-2, anti-IFN, granzyme-A, and perforin specific-antibodies (miltenyiBiotech, Germany). All antibodies were titrated before use.

**Fig. 1:**
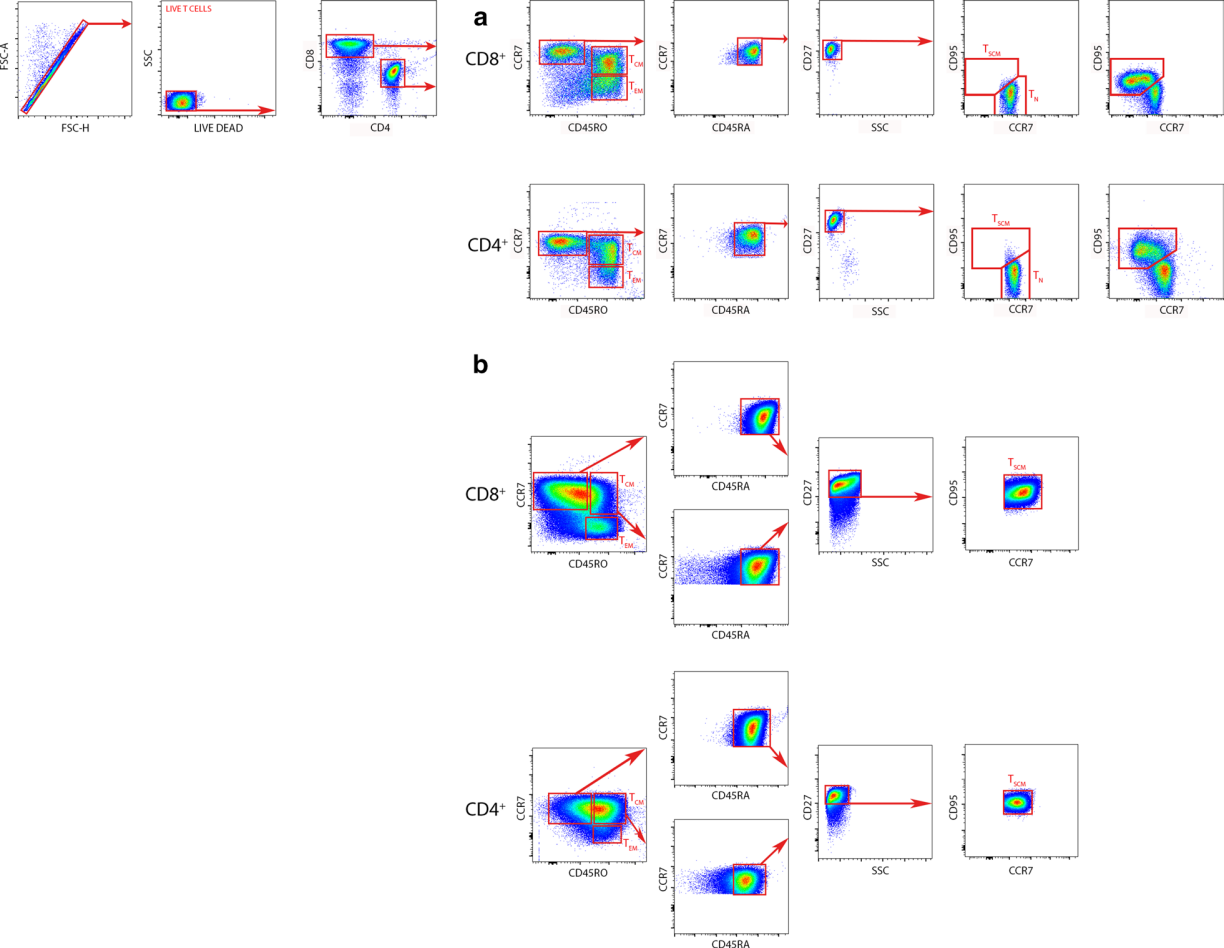
Gating strategy for the identification of human T_SCM_. T cells were identified by gating on lymphocytes (SSC versus FSC), singlets (FSC-H versus FSC-A) and live T cells (SSC versus LIVE/DEAD). CD4^+^ and CD8^+^ T cells were simultaneously identified with anti-CD4 and anti-CD8 antibodies. **a** Gating strategy of fresh blood cells. After gating on CD4^+^ and CD8^+^ cells, T_CM_ and T_EM_ subpopulations were identified based on CCR7 and CD45RO expression. In the gated CCR7^+^CD45RO^−^ population, cells expressing CD45RA and CD27 were further analyzed. In this later population (CCR7^+^CD45RO^−^CD45RA^+^CD27^+^), T_N_ and T_SCM_ were identified based on the CD95 expression. T_N_ is defined as CCR7^+^CD45RO^−^CD45RA^+^CD27^+^CD95^−^ whereas T_SCM_ subpopulation is defined as CCR7^+^CD45RO^−^CD45RA^+^CD27^+^CD95^+^. *Red arrows* indicate the sequential gating strategy. **b** Gating strategy of 10 days culture cells. After gating on CD4^+^ and CD8^+^ cells, T_CM_ and T_EM_ subpopulations were identified based on CCR7 and CD45RO expression. In the gated CCR7^+^CD45RO^−^ population, cells expressing CD45RA and CD27 were further analyzed. In this later population (CCR7^+^CD45RO^−^CD45RA^+^CD27^+^), T_SCM_ were identified based on the CD95 expression. T_SCM_ subpopulation is defined as CCR7^+^CD45RO^−^CD45RA^+^CD27^+^CD95^+^. Similarly, in the gated CCR7^+^CD45RO^+^ population, cells expressing CD45RA, CD27 and CD95^+^ identify a T_SCM-_like subpopulation, which is defined as CCR7^+^CD45RO^+^CD45RA^+^CD27^+^CD95^+^. *Red arrows* indicate the sequential gating strategy

Corresponding isotypes for each antibody were used as controls. Cells were analyzed by flow cytometry using a MACS Quant cytometer (Miltenyi Biotech, Germany) and the FlowJo version V10 software (Treestar, USA). Different T cell subpopulations obtained from fresh blood (Fig. 1a) and cultured cells (Fig. 1b) were determined by a previous published gating strategy [11] using fluorescence minus one (FMO) controls for each antibody (i.e., samples stained with all fluorochromes except the one of interest). Particularly, T_SCM_ were identified by a previously described plotting strategy [11]. Briefly, CD4^+^ and CD8^+^ cells were sorted and a CCR7 against CD45RO plot were used to select CCR7^+^CD45RO^−^ cells in each subpopulation. In these cells, we identified CCR7^+^CD45RO^−^CD45RA^+^ cells by plotting CCR7 against CD45RA. Then, CD27 was used to detect CCR7^+^CD45RO^−^CD45RA^+^CD27^+^ cells. Finally, a plot of CD95 against CCR7 was used to identify CCR7^+^CD45RO^−^CD45RA^+^CD27^+^CD95^+^ T_SCM_ (Fig. 1).

### Statistical analysis

Statistical analysis was performed with GraphPad Prism 6 (GraphPad Software, USA). Data are shown as the mean ± SEM. Differences were tested for statistical significance using one-way ANOVA test. A p value <0.05 was considered significant.

## Results

### Short CD3/CD28 costimulation enriches for memory stem T cells (T_SCM_) cultured with IL-7/IL-15

**Fig. 2:**
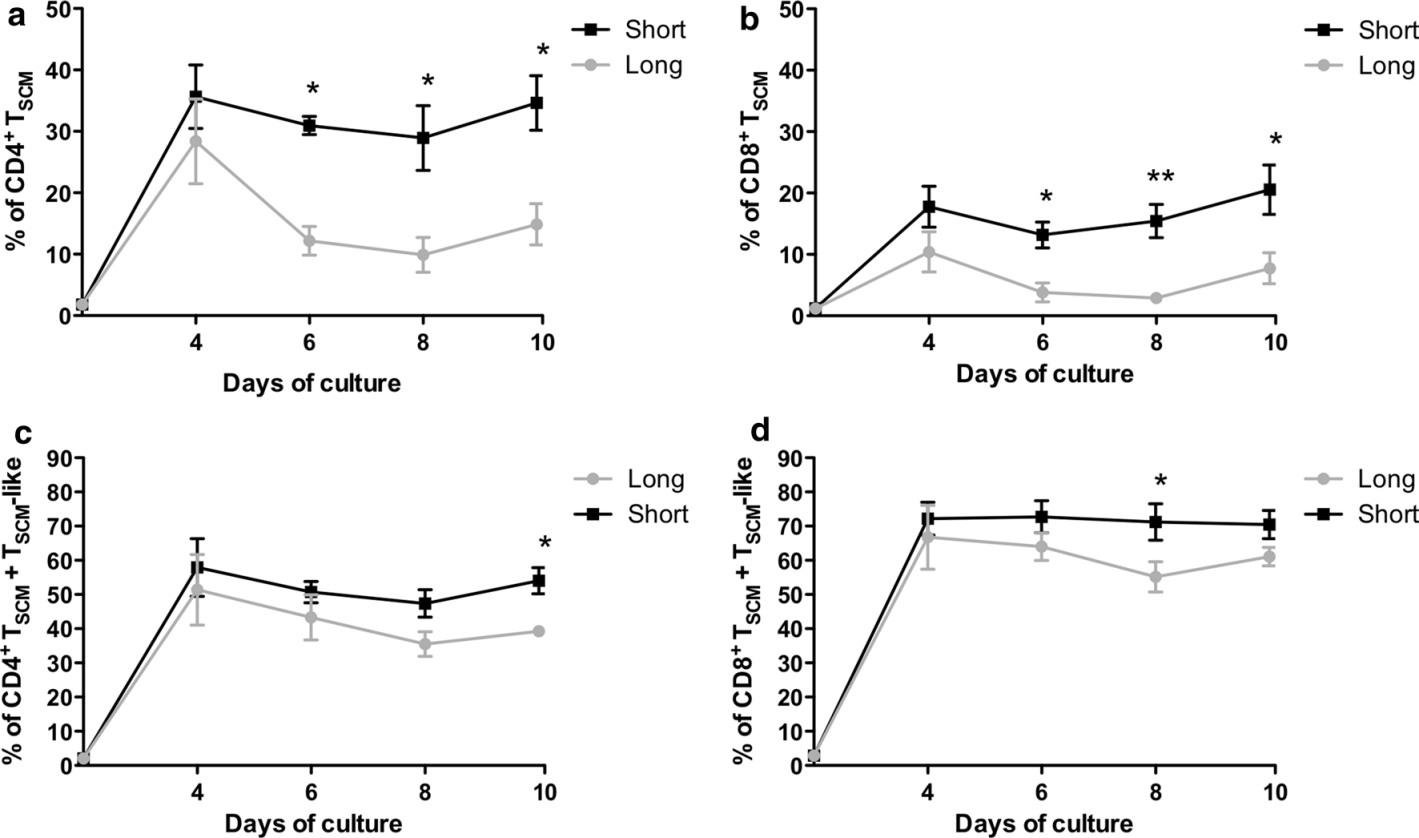
Short CD3/CD28 costimulation increases CD4^+^ and CD8^+^ T_SCM_ frequencies compared with long costimulation. Naïve T cells from healthy donors (n = 6) were cultured for 10 days with short (48 h) (*solid black line*) or long (*solid grey line*) costimulation. **a**, **b** Frequency of CD4^+^ (**a**) and CD8^+^ (**b**) T_SCM_ cell subset (mean ± SEM). **c**, **d** Frequencies of total T_SCM_ (T_SCM_ + T_SCM_-like) CD4^+^ (**c**) and CD8^+^ (**d**) (mean ± SEM). *p < 0.05; **p < 0.01; ***p < 0.001

To assess whether the length of CD3/CD28 costimulation has an impact on the maintenance of the T_SCM_ phenotype in vitro, naïve T cells were cultured with low doses of IL-7 and IL-15 and activated with magnetic beads coated with anti-CD3/anti-CD28. A short CD3/CD28 costimulation (48 h) was compared to a long stimulus (the entire period of cell culture: 10 days) by analyzing individual T-cell subsets at different time points. As shown in Fig. 2a, while the percentage of CD4^+^ T_SCM_ at day 4 was comparable between both conditions (35.64 ± 5.1 % and 28.38 ± 6.9 %; p = 0.42), the short CD3/CD28 costimulation led to a significant increase in the frequencies of CD4^+^ T_SCM_ after day 4 that was maintained until the end of the culture (34.6 ± 4.4 % vs 15.6 ± 4.24 % respectively; p = 0.008) (Fig. 2a).

Similar results were obtained in the CD8^+^ T_SCM_ population (Fig. 2b). A short costimulation generated a significant increase of CD8^+^ T_SCM_ frequencies compared to a long costimulation (20.5 ± 4.00 % vs 7.7 ± 2.53 % at day 10, respectively; p = 0.02). Day 10 was selected as an endpoint for culture since a decline in T_SCM_ numbers was observed after this time (data not shown) and over this time period T_SCM_ expand to numbers considered to be sufficient for clinical translation.

According to previous data [9], when T_SCM_ are cultured in vitro they may also acquire the expression of CD45RO, while preserving CD45RA and CCR7^+^CD27^+^CD95^+^ expression (i.e., a T_SCM_-like phenotype). We found no differences in the percentage of both CD4^+^ and CD8^+^ T_SCM_-like cells across different time points over the culture period (19.4 ± 3.06 % vs 24.4 ± 2.6 % in CD4^+^; p = 0.252 and 49.95 ± 3.6 % vs 53.36 ± 1.04 % in CD8^+^; p = 0.35).

When total T_SCM_ (i.e., T_SCM_ + T_SCM_-like population) were analyzed (Fig. 2c, d), a higher percentage was observed in the CD4^+^ population after a short stimulation, reaching 54.02 ± 3.837 % at day 10 vs 38.49 ± 1.48 % in the long stimulus condition (p = 0.0054) (Fig. 2c). In the CD8^+^ population, a trend to higher percentages were found after short costimulation compared to a long costimulation (70.45 ± 4.1 % vs 60.2 ± 3.29 %; p = 0.08) (Fig. 2d).

### IL-21 increases the frequencies of T_SCM_ generated under short CD3/CD28 costimulation

**Fig. 3:**
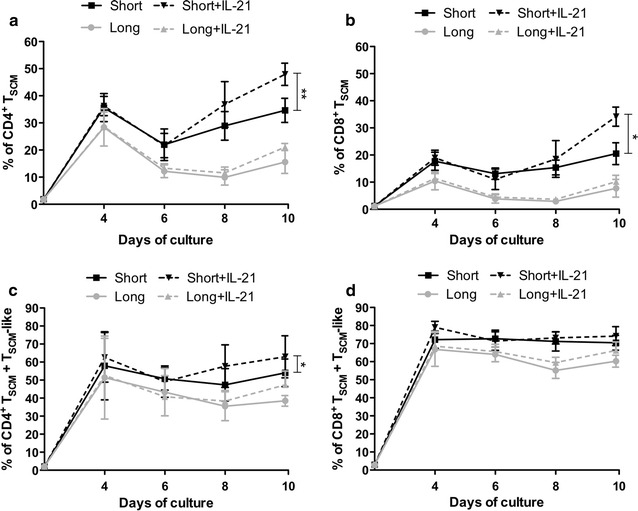
IL-21 enhances CD4^+^ and CD8^+^ T_SCM_ frequencies. Naïve T cells from healthy donors (n = 6) were cultured for 10 days with short (*solid black line*) or long (*solid grey line*) costimulation and in the presence (*black dashed line*) or absence (*grey dashed line*) of IL-21. **a**, **b** Frequencies of CD4^+^ and CD8^+^ T_SCM_ (mean ± SEM) are shown in (**a**) and (**b**), respectively. **c**, **d** Frequencies of CD4^+^ and CD8^+^ Total T_SCM_ (T_SCM_ + T_SCM_-like) (mean ± SEM) are shown in (**c**) and (**d**), respectively. *p < 0.05; **p < 0.01; ***p < 0.001

Next, we analyzed the impact of IL-21 on the in vitro generation and maintenance of T_SCM_. As shown in Fig. 3a, the addition of IL-21 to the culture from day 0 significantly increased the percentage of CD4^+^ T_SCM_ after a short CD3/CD28 costimulation (47.92 ± 4.10 % vs 34.62 ± 4.44 %; p = 0.006). However, IL-21 had no impact on the percentage of CD4^+^ T_SCM_ cultured under long costimulation (21.1 ± 1.31 % vs 15.6 ± 4.24 %; p = 0.17) (Fig. 3a). The same hold true for CD8^+^ T_SCM_. IL-21 significantly increased the percentage of CD8^+^ T_SCM_ cultured under short costimulation (34.15 ± 3.51 % vs 20.5 ± 4.005 %; p = 0.00039) at day 10 of culture, while adding IL-21 to the long costimulation condition resulted in no significant changes (10.20 ± 2.34 % vs 7.73 ± 3.27 %; p = 0.14) (Fig. 3b).

Finally, we analyzed if IL-21 could increase the percentage of total T_SCM_ (i.e., T_SCM_ + T_SCM_-like population). At day 10, significant higher frequencies were noted in the CD4^+^ subset in the short costimulation in the presence of IL-21 (62.92 ± 5.83 % vs 54.02 ± 3.83 %; short + IL-21 vs short, respectively; p = 0.025) (Fig. 3c). Although the differences between short and long stimulation in the CD8^+^ subset are enhanced by the addition of IL-21 (74.15 ± 5.26 % vs 60.2 ± 3.29 %; short + IL-21 vs short respectively; p = 0.04), no significant differences were observed in the short stimulation condition by the addition of IL-21 regarding total T_SCM_ (74.15 ± 5.26 % vs 70.45 ± 4.13 %; p = 0.56) (Fig. 3d).

**Fig. 4:**
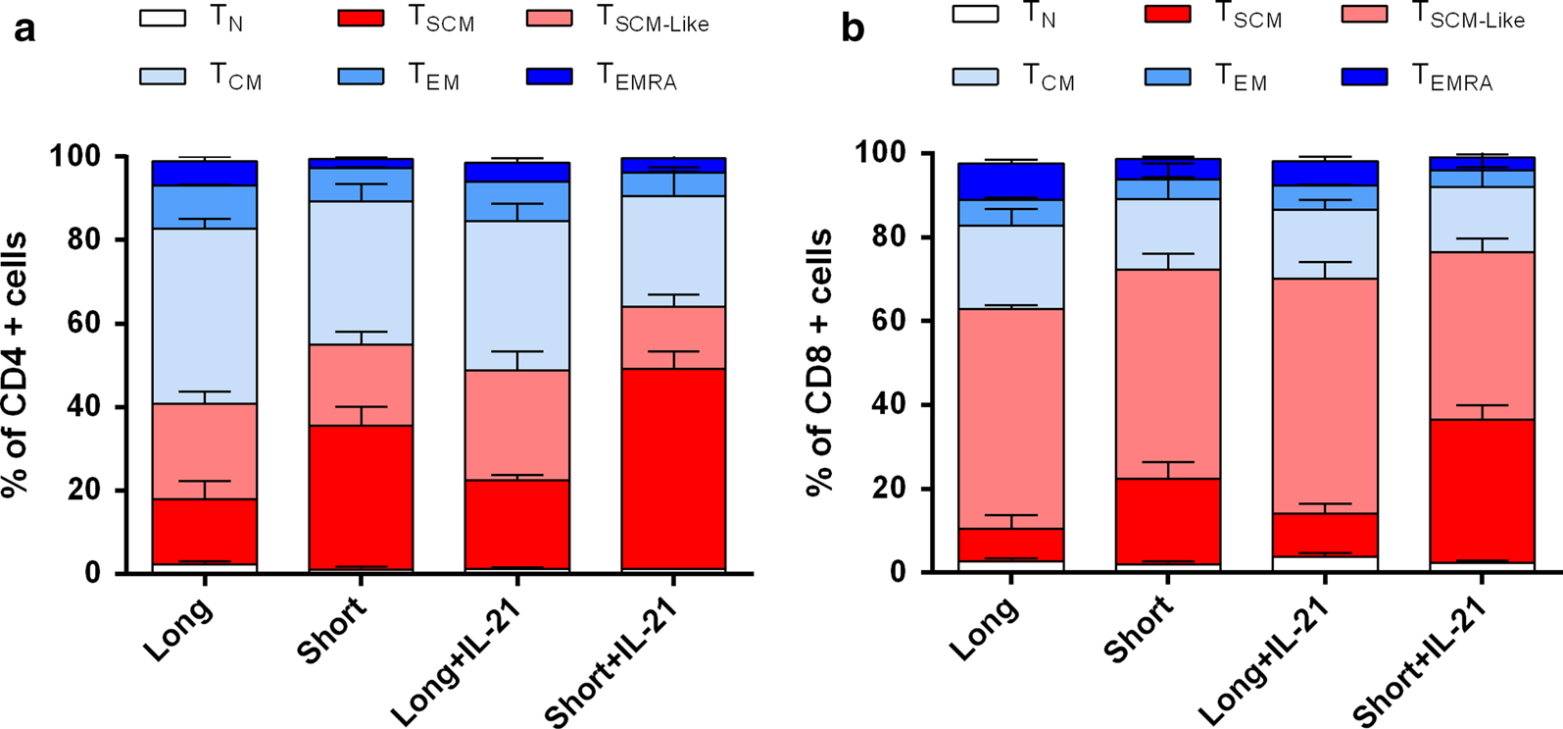
Analysis of CD4^+^ and CD8^+^ T cell subsets composition. **a**, **b** Column graphs showing the relative frequencies (mean ± SEM) of CD4^+^ (**a**) and CD8^+^ (**b**) T cell subsets respectively, for each condition tested (long costimulation, short costimulation, long + IL-21, and short + IL-21). T_N_ (T Naïve), T_CM_ (T central memory), T_EM_ (T effector memory), T_EMRA_ (T effector memory-RA+ cells)

IL-21 is a cytokine with action on multiple T cell subsets. To clarify if the short stimulus with IL-21 could affect any of the populations present in the culture, different memory T cell subsets were analyzed at day 10 (Fig. 4). The percentages of central memory T cells were 34.27 ± 4.13 % vs 26.4 ± 6.9 %; p = 0.95 in CD4^+^ and 16.6 ± 8.52 % vs 15.45 ± 9.5 %; p > 0.05 in CD8^+^ (short vs short + IL-21 respectively). The percentages of effector memory T cells were 7.97 ± 0.12 % vs 5.54 ± 0.07 %; p > 0.05 in CD4^+^, and 4.8 ± 0.54 % vs 4.15 ± 0.59 %; p > 0.05 in CD8^+^ (short vs short + IL-21 respectively). In addition, CD4^+^/CD8^+^ T_SCM_ ratio was also analyzed to explore the possibility of an overexpansion of CD8^+^ in detriment of CD4^+^. IL-21 did not preferentially enriched for the CD8^+^ population since the CD4^+^ cells were maintained over the cell culture period in all the conditions tested (CD4^+^/CD8^+^ T_SCM_ ratio: 1.03 ± 0.28 vs 1.34 ± 0.19 for long versus long + IL-21 costimulation, and 0.68 ± 0.14 vs 0.72 ± 0.002 for short vs short + IL-21 costimulation; p = 0.23).

### Short costimulation combined with IL-21 enhance T_SCM_ expansion

**Fig. 5:**
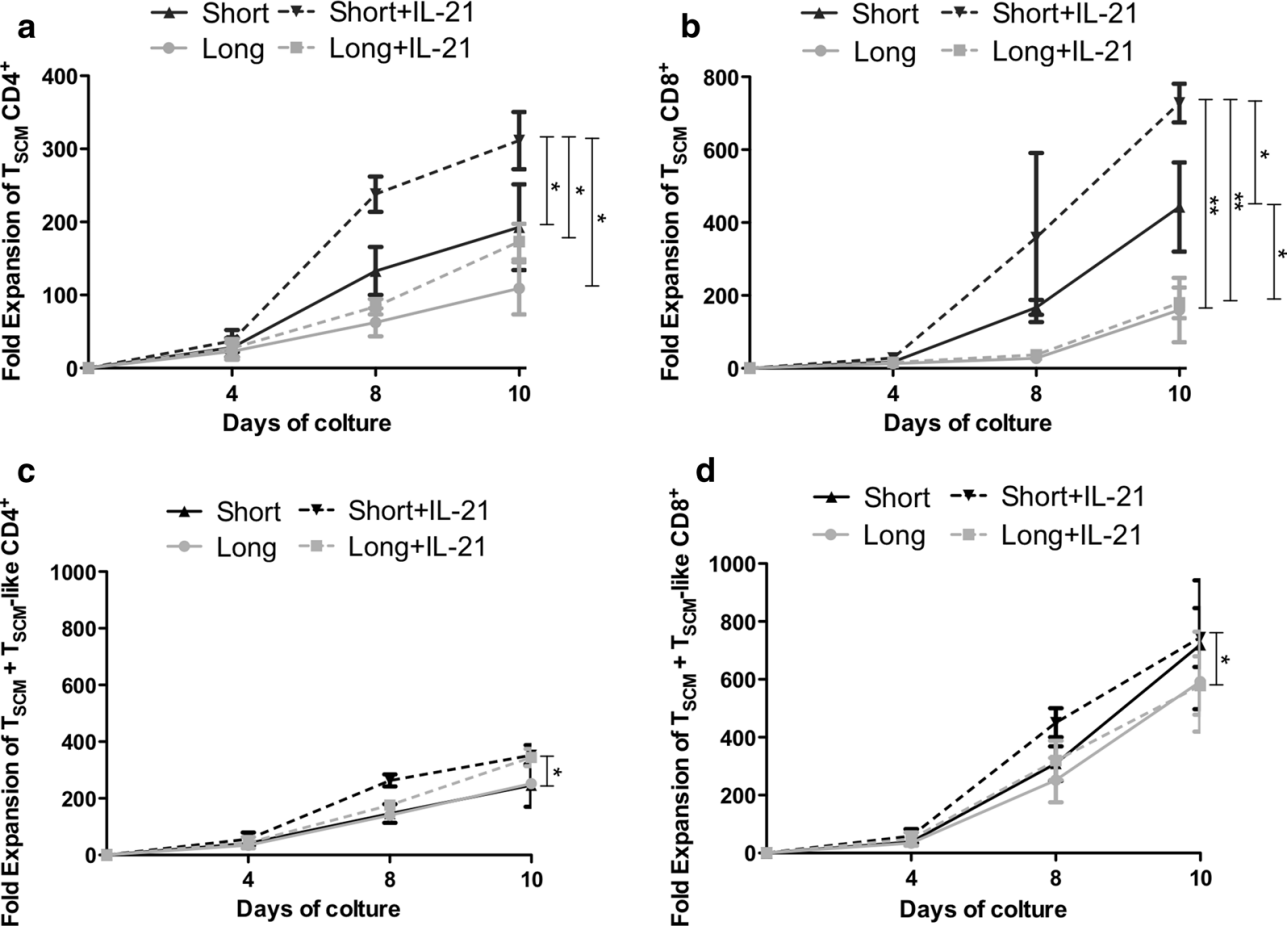
IL-21 enhances CD4^+^ and CD8^+^ T_SCM_ expansion. Naïve T cells from healthy donors (n = 3) were cultured for 10 days and fold expansion were analyzed in short costimulation (*solid black line*), long costimulation (*solid grey line*) short + IL-21 (*black dashed line*) and long + IL-21 (*grey dashed line*) conditions. **a**, **b** Absolute fold expansion of CD4^+^ (**a**) and CD8^+^ (**b**) (mean ± SEM), expressed in increment of cells from the starting culture time point is shown. **c**, **d** Absolute fold expansion of CD4^+^ (**c**) and CD8^+^ (**d**) total TSCM (TSCM + TSCM-like) (mean ± SEM). *p < 0.05; **p < 0.01; ***p < 0.001

Next, we analyzed the effect of IL-21 in T_SCM_ expansion under the costimulation conditions described. As shown in Fig. 5a, b, the addition of IL-21 to the culture produced a significant increase in the T_SCM_ expansion. The combination of short costimulation and IL-21 led to the greater T_SCM_ expansion, for both CD4^+^ and CD8^+^ subsets, among all conditions tested (CD4^+^ T_SCM_: 311.34 ± 39 fold vs 192.84 ± 58.61 fold; T_SCM_: 728.06 ± 53 fold vs 442.73 ± 122 fold; IL-21 vs no IL-21 respectively; p = 0.040) (Fig. 5a, b). Finally, we analyzed the impact of IL-21 on total T_SCM_ expansion. As shown in Fig. 5c, d, the combination of short costimulation and IL-21 led to the greatest expansion of total CD4^+^ and CD8^+^ T_SCM_ (351.79 ± 36 fold vs 251.83 ± 16.6 fold; p = 0.07, and 744.46 ± 101.76 fold vs 592.3 ± 172 fold; short + IL-21 vs long, respectively; p = 0.1) (Fig. 5c, d).

### T_SCM_ shows different functional properties

To further define the functional features of T-cells generated under these conditions, we analyzed the expression of IL-2, IFN-γ, granzyme-A and perforin on FACS-purified T_SCM_, T_CM_ and T_EM_ at day 10 of culture. We found that both CD4^+^ and CD8^+^ T_SCM_ secrete higher levels of IL-2 compared to T_CM_ (CD4: 84.65 ± 0.75 % vs 83.3 ± 0.7 %; p = 0.48; CD8: 68.86 ± 1.9 % vs 62.56 ± 0.13 %, p = 0.03) and T_EM_ (CD4: 84.65 ± 0.75 % vs 80.5 ± 0.75 %; p = 0.057; CD8: 68.86 ± 1.9 % vs 57.8 ± 1.12 %, p = 0.0022) (Additional file 1: Figure S1 A, B). Moreover, we observed lower proportion of CD4^+^ and CD8^+^ T_SCM_ that secrete IFN-γ compared to T_CM_ (CD4: 8.18 ± 0.16 % vs 13.6 ± 1 %; p = 0.016; CD8: 18.66 ± 4.2 % vs 48.76 ± 8.2 %, p = 0.0397) and T_EM_ (CD4: 8.18 ± 0.16 % vs 21.55 ± 0.25 %; p = 0.0012; CD8: 18.66 ± 4.2 % vs 60.86 ± 6.4 %, p = 0.0091) and granzyme-A compared to T_CM_ (CD4: 34.83 ± 8.6 % vs 55.66 ± 1.53 %; p = 0.0631; CD8: 7.2 ± 3.3 % vs 75.8 ± 0.3 %, p = 0.0004) and T_EM_ (CD4: 34.83 ± 8.6 % vs 78.13 ± 2.36 %; p = 0.0033; CD8: 7.2 ± 3.3 % vs 78.1 ± 0.5 %, p = 0.0004). We also observed that CD4^+^ and CD8^+^ T_SCM_ stained mainly negative for perforin (0.024 ± 0.002 % and 0.9 ± 0.3 %, respectively) (Additional file 1: Figure S1 C–H).

Finally, we analyzed the phenotype and self-renewal capacity of FACS-purified T_SCM_ cells after being expanded for 10 days. We observed that, in contrast to T_CM_ and T_EM_ subsets, both CD4^+^ and CD8^+^ T_SCM_ maintained their phenotype and proliferative capacity in culture for another 10 days after sorting. Remarkably, 93 % of the CD4^+^ and 88 % of CD8^+^ T cells displayed a T_SCM_ phenotype after 20 days in culture with IL-7, IL-15 and IL-21, while T_CM_ and T_EM_ largely lost their proliferative capacities and became differentiated (Additional file 2: Figure S2).

### CD4^+^ and CD8^+^ T_SCM_ are efficiently transduced by lentiviral vectors

**Fig. 6:**
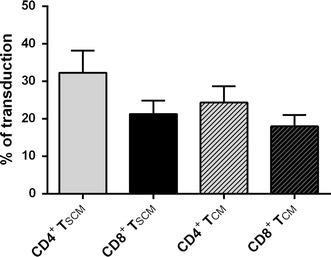
Analysis of transduction efficiency with a GFP-expressing lentivirus. Lentivirus transduction efficiency measured in CD4^+^ and CD8^+^ T_SCM_ and T_CM_ subsets by flow cytometry (% of cells expressing GFP; mean ± SEM)

Unlike activated and dividing cells, quiescent T cells are less permissive to vector transduction. To assess whether T_SCM_ generated under this new conditions could be genetically modified for adoptive immunotherapy, an encoding-GFP lentivirus was used to transduce T_SCM_. We have analyzed transduction efficiencies (multiplicity of infection (m.o.i.) = 1) following stimulation of naïve T cells for 48 h with magnetic anti-CD3/CD28 beads and IL-7, IL-15 and IL-21. Gene transfer in CD4^+^and CD8^+^ cells averaged 28 and 19 % respectively. The transduction efficiency was also analyzed in the different memory subset presents in the culture. We observed similar transduction percentages of T_SCM_ and T_CM_ in both CD4^+^ and CD8^+^ population (32.25 ± 5.91 % vs 24.30 ± 4.36 % in CD4^+^, T_SCM_ vs T_CM_ respectively; P = 0.056 and 21.29 ± 3.54 % vs 17.96 ± 3.07 % in CD8^+^, T_SCM_ vs T_CM_ respectively; p = 0.5;) (Fig. 6).

## Discussion

The generation of T_SCM_ for ACT has become a clinically relevant issue since preclinical studies have shown that the use of the less-differentiated T_SCM_ is associated with superior T-cell engraftment, persistence, and antitumor efficacy [3–6]. Although ACT to treat cancer have experimented significant progress in the past few years, particularly with the chimeric antigen receptor (CAR) approach, improvements on their clinical efficacy are clearly needed. Remarkably, although no ACT clinical trials with T_SCM_ have been done in cancer patients so far, studies in patients with lymphoma receiving CAR-T cells showed that the infusion of >5 % of cells with a T_SCM_ phenotype correlated with greater persistence and expansion in vivo, properties associated with clinical efficacy [20].

Previous studies have shown that the combination of IL-7, IL-15 and CD3/CD28 costimulation led to the generation of T_SCM_ from naïve T cells [9]. Interestingly, cells generated under this condition also express CD45RO, which indicates that the combination of prolonged CD3/CD28 costimulation with IL-7 and IL-15 induce a more differentiated T_SCM_ (T_SCM_-like) than the originally described [9]. This could be related to the stimulation through CD3 and CD28, which expands T cells efficiently but also causes a drastic change in the cell phenotype, including downregulation of CD45RA, CCR7 and CD62L [6, 13, 21]. Although both subsets have comparable gene expression profiles, it remains to be proven that the T_SCM_-like subset (i.e., those cells expressing CD45RO in addition to CD45RA and CCR7) retain similar antitumor efficacy than the less differentiated T_SCM_. In this regard, when CAR-T cells were cultured with IL-7/IL-15, only the fraction CD45RA^+^/CCR7^+^ retained the ability to expand after repetitive antigen stimulations [20]. Accordingly, strategies directed to preserve the phenotype of T_SCM_ during their ex vivo generation should be regarded a clinical priority.

In this study, we show that a very short (48 h) CD3/CD28 costimulation increases the percentage of T_SCM_ within the CD4^+^ and CD8^+^ subsets in detriment of a more differentiated phenotypes. Moreover, the contribution of the less differentiated T_SCM_ to the total T_SCM_ frequency is superior in the short costimulation condition compared to a long costimulation, in line with the concept that prolonging the CD3/CD28 stimulation in the culture is associated with T cell differentiation [21, 22]. In our condition, T_SCM_ proliferation was not affected by the absence of a prolonged CD3/CD28 signal, suggesting that a continued costimulatory signal would not be necessary to promote an efficient ex vivo T_SCM_ expansion in the presence of IL-7 and IL-15 [23–25].

The T-cell receptor signal strength impacts on T cell expansion and differentiation [26]. Methods for the generation of effector T cells based on CD3/CD28 stimulation have relied on the use of a high bead:cell ratio (e.g., 3:1). We observed that a high bead-to-cell ratio reduced the frequency of T_SCM_ and did not generate a strong T cell proliferative response, a fact that may be associated with activation-induced T cell death [21, 27]. In contrast, in our hands, a low bead-to-cell ratio (e.g., 1:2) coupled to a short costimulation was the best condition for promoting the generation of higher T_SCM_ frequencies while maintaining their proliferative capacity. This is in line with recent studies showing that low or equal bead:cell ratios give the higher yields of T cells when cultured in IL-7 and IL-15 [21]. Moreover we observed that the short TCR triggering do not reduced the transduction efficiency of T_SCM_, probably due to the presence of the IL-7, IL-15 or IL-21 cytokines, which promote long-term survival of resting T cells, while maintaining their maturational status [28].

Previous studies have shown that IL-21 synergizes with either IL-7 or IL-15 to regulate CD8^+^ T cell expansion and to maintain a memory phenotype [18, 19, 29]. However, the potential role of IL-21 in the ex vivo generation of T_SCM_ has not been described. Here, we show for the first time that the addition of IL-21 significantly increases the percentage of CD4^+^ and CD8^+^ T_SCM_ cultured with a short CD3/CD28 stimulation and IL-7/IL-15. Remarkably, addition of IL-21 to that condition further increased the proliferation rate of T_SCM_, which translated into an increase of absolute numbers of T_SCM_ at the end of the culture. Although previous studies have shown that CD3/CD28 costimulation in combination with IL-7 and IL-15 promote a substantial increment in T_SCM_ expansion [9], the reduction in the length of costimulation and, particularly, the addition of IL-21 produces even higher numbers of T_SCM_ that could be sufficient for clinical translation. Interestingly, this effect was noted in both CD4^+^ and CD8^+^ T_SCM_, and although the proliferation rate was greater in the CD8^+^ subset compared to that in CD4^+^, it is remarkable that the CD4^+^/CD8^+^ ratios of T_SCM_ were preserved over the culture period.

According to previous studies showing a higher expression of IL-21 receptor in activated naïve T cells [30], and that the effect of IL-21 might depend on the T cell differentiation status [21, 31], we observed that the impact of IL-21 was more pronounced in those T cells with a phenotype close to naïve T cells, resulting in an enrichment of less differentiated T_SCM_ within the total T_SCM_ subset. Moreover, we have noticed a higher expression of IL-21R in naïve and T_SCM_ cells compared to more differentiated T cells subsets (57.5 % of T_SCM_ vs 22 % of T_CM_ in CD4^+^ and 42.6 % of T_SCM_ vs 5 % of T_CM_ in CD8^+^) (data not shown). Interestingly, this difference is more pronounced at day 4 of the culture (in particular in CD8^+^ T cells), which could increase the effect of IL-21 in the following days.

It has been shown that self-renewal depends on STAT3 (Signal transducer and activator of transcription 3) signaling cytokines, including IL-6, IL-10 and IL-21, which inhibit exhaustion and differentiation of T cell [32]. Similarly, T cells expanded in the presence of IL-21 (the unique common γ-chain cytokines to sustain STAT3 activation) prevent the loss of CD45RA, CD62L, CD27, and IL-7Rα, increasing the ability of the cell to release IL-2 [33, 34]. Interestingly, we noticed that T_SCM_, T_CM_ and T_EM_ cultured in the presence of IL-21 express high level of IL-2. In addition, T_SCM_ express low levels of IFN-γ, granzyme-A and perforin compared to T_CM_ and T_EM_. Thus, T_SCM_ display phenotypic and functional characteristics of early-differentiated cells.

We also observed that highly purified naïve T cells are a more suitable starting population for T_SCM_ generation in contrast to the use of “bulk” peripheral blood mononuclear cells [10, 20]. The use of purified naïve T cells may overcome the variability of this subset observed between donors when using whole peripheral blood lymphocytes. Another important issue is the potential deleterious effect that more differentiated memory T cells present in the starting cell population may have on the generation of T_SCM_. Accordingly, in our experience, T_SCM_ are more efficiently generated when naïve T cells are used as starting population (data not shown), in line with previous data from Bonini´s group [9].

## Conclusions

In summary, we demonstrated that when naïve T cells are cultured with IL-7 and IL-15, the use of a very short CD3/CD28 costimulation enhances CD4^+^ and CD8^+^ T_SCM_ generation and expansion. Moreover, the addition of IL-21 to this culture conditions produces a further enrichment and greater expansion of CD4^+^ and CD8^+^ T_SCM_. Importantly, the conditions described here allow an efficient transduction of both CD4^+^ and CD8^+^ T_SCM_ with lentivirus. These refinements on the in vitro conditions may be of clinical relevance for the generation of T_SCM_ for ACT of patients with cancer, and can be easily adapted to preserve this cell subset in the final product under GMP conditions.

## Additional files

10.1186/s12967-016-0973-y T_SCM_ functional properties. A-F Frequencies of CD4^+^ and CD8^+^ T_SCM_, T_CM_, and T_EM_ cytokine-producing cells (mean ± SEM). Quantitation of IL-2 (A), IFN-γ (C), granzyme-A (E) and perforin (G) production by CD4^+^ cells and IL-2 (B), IFN-γ (D), granzyme-A (F) and perforin (H) production by CD8^+^ cells. *p < 0.05; **p < 0.01; ***p < 0.001.
10.1186/s12967-016-0973-y Long term culture and proliferative capacity of T_SCM_ cells. Isolated naïve T cells were cultured under IL-7, IL-15, IL-21 and short costimulation until day 10. Selected T_SCM_ were sorted and cultured for another 10 days. Column graphs showing the relative frequencies of CD4^+^ and CD8^+^ T cell subsets from day 0 to day 10 after T_SCM_ sorting (total duration of culture is 20 days).

## Abbreviations

ACT: adoptive T cell therapy
T_CM_: central memory T cells
CAR: chimeric antigen receptor
T_EM_: effector memory T cells
T_EMRA_: effector memory-RA + T cells
FMO: fluorescence minus one
T_SCM_: memory stem T cells
m.o.i: multiplicity of infection
T_N_: naïve T cells
PBMCs: peripheral blood mononuclear cells
γc: γ-chain
IFN-γ: interferon gamma
STAT3: signal transducer and activator of transcription 3
