# Supplementary figures and images for: A short CD3/CD28 costimulation combined with IL-21 enhance the generation of human memory stem T cells for adoptive immunotherapy
Source: J Transl Med. 2016 Jul 19;14:214. doi: 10.1186/s12967-016-0973-y (PMC4952071; doi:10.1186/s12967-016-0973-y)

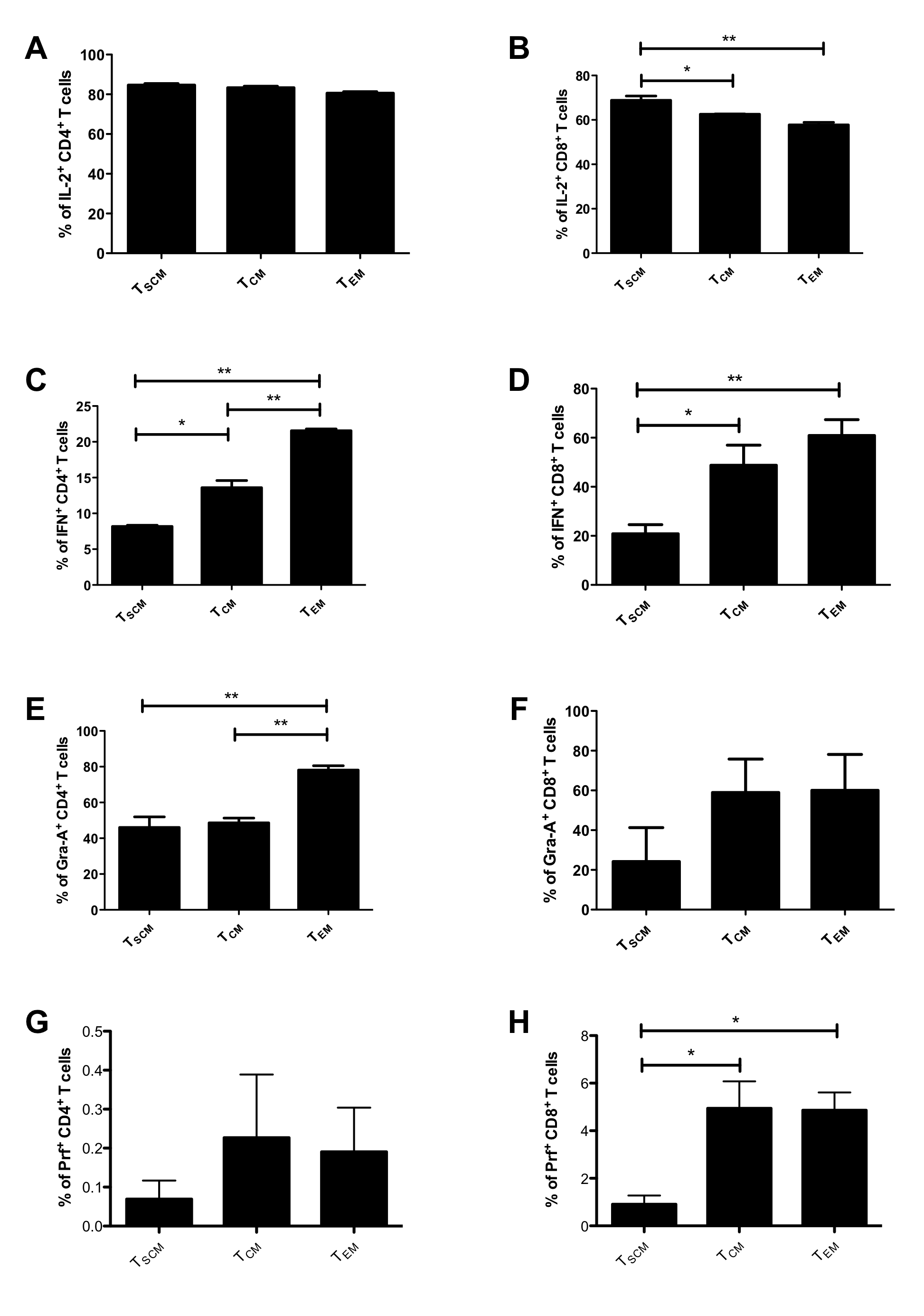

Supplement: Supplementary file 1 — 10.1186/s12967-016-0973-y TSCM functional properties. A-F Frequencies of CD4+ and CD8+ TSCM, TCM, and TEM cytokine-producing cells (mean ± SEM). Quantitation of IL-2 (A), IFN-γ (C), granzyme-A (E) and perforin (G) production by CD4+ cells and IL-2 (B), IFN-γ (D), granzyme-A (F) and perforin (H) production by CD8+ cells. *p < 0.05; **p < 0.01; ***p < 0.001. [file 12967_2016_973_MOESM1_ESM.tiff]
